# Identification of novel immune-related biomarker and therapeutic drugs in Parkinson disease via integrated bioinformatics analysis

**DOI:** 10.1097/MD.0000000000034456

**Published:** 2023-08-04

**Authors:** Xiaoxia Yang, Zhiyun Wang

**Affiliations:** a Department of Neurology, Tianjin First Central Hospital, Nankai District, Tianjin, China.

**Keywords:** candidate drugs, diagnostic model, immune-related genes, Parkinson disease, WGCNA

## Abstract

The present study was designed to identify immune-related biomarker and candidate drugs for Parkinson disease (PD) by weighted gene co-expression network analysis.

**Methods:**

Differentially expressed genes were identified in PD and healthy samples in the Gene Expression Omnibus (GEO) database. Besides, immune-related genes were obtained from the immunology database. Then, a co-expression network was constructed by the weighted gene co-expression network analysis package. Diagnostic model for PD was constructed by Lasso and multivariate Cox regression. Furthermore, differentially expressed genes (DEGs) were used to establish PPI and competing endogenous RNA (ceRNA) networks. Functional enrichment and pathway analysis were performed. Drug-hub gene interaction analysis was performed via DGIdb database.

**Results:**

PD samples and normal samples were found to have 220 upregulated genes and 216 downregulated genes in the GSE6613 dataset. The differentially expressed genes contained 50 immune-related genes, with 40 upregulated genes and 10 downregulated genes. We obtained 7 hub genes by intersecting the DEGs and candidate hub genes. As potential diagnostic markers, 2 immune-related DEGs were identified among the 7 hub genes. According to functional enrichment analysis, these DEGs were mainly enriched in immune response, inflammatory response, and cytokine-cytokine receptor interactions. Totally, we obtained 182 drug-gene interaction pairs in Drug-Gene Interaction database (DGIdb).

**Conclusion:**

Our results revealed crucial genes and candidate drugs for PD patients and deepen our understanding of the molecular mechanisms involved in PD.

## 1. Introduction

Parkinson disease (PD) is a general progressive geriatric neurodegenerative disease that mainly affects the movement and health of the elderly. The features of Parkinson disease contain resting tremor, stiffness, bradykinesia, and muscular rigidity.^[1,2]^ Worldwide, approximately 7 million people are affected by PD, which is characterized by tremors and slow movement.^[3]^ The etiology of PD remains unclear, involving complex interactions between genetic and environmental factors.^[4]^ Because of this disorder complex pathogenesis, it is mainly treated by symptomatic measures such as the replacement of dopamine.^[5]^ In recent years, molecular biomarkers were demonstrated highly useful as clinical tools for PD diagnosis.^[6]^ Thus, it is imperative to find novel methods for early detection and intervention of treatment for better outcomes.

Parkinson disease is associated with immune mechanisms, especially α-synuclein mediated immune cell activation. In general, α-synuclein is a functional protein that is mainly responsible for vesicle trafficking and neurotransmitter secretion. In PD, the accumulation of α-synuclein makes it a self-antigen and elicits an autoimmune response in immune cells. These evidences suggest that the pathology of PD are closely related to immunology, and PD may be an autoimmune response-mediated disease.^[7]^ In-depth research on peripheral immune changes in PD patients has gradually attracted people’s attention. Immune dysfunction is associated with genetic forms of the disease and genetic risk factors, such as HLA and α-synuclein (SNCA).^[8,9]^ The pathological process of PD can destroy the blood-brain barrier, and the destruction of the blood-brain barrier leads to increased infiltration of peripheral immune cells into the central nervous system, which has been identified as one of the main contributing factors of PD.^[10]^ Increased peripheral immune cell infiltration may cause excessive microglial inflammation, oxidative stress, and cytotoxicity, thereby exacerbating neurodegeneration in PD. Taken together, current evidence suggests that the peripheral immune system is closely related to the progression of PD.

The mechanisms of PD at the molecular level are critically important for treating the disease. With the wide application of microarray technology and RNA-seq technology, PD-related genes have been widely identified, which is an important step in exploring the complex pathology of PD and developing drugs that combat the illness. Gene co-expression networks are commonly used for identification of gene modules in a set of samples including PD. The weighted gene co-expression network analysis (WGCNA) is a method of bioinformatics that reveals correlations between genes in various samples. In the present study, differentially expressed genes (DEGs) between PD patient and healthy samples were identified in GEO microarray. WGCNA was used to screen the key immune-related modules for PD. Then we used the immune-related hub gene to construct a Parkinson diagnostic model. Finally, we performed drug-gene interaction analysis to predict candidate drugs for PD.

## 2. Materials and methods

### 2.1. Data collection and preprocessing

Expression profiles of PD (GSE6613 and GSE72267) were downloaded from the Gene Expression Omnibus database (Table 1). The GSE6613 dataset included 50 PD whole blood samples and 22 healthy samples on the GPL96 platform. The GSE72267 dataset was composed of 40 PD whole blood samples and 19 control samples. Gene expression profiles were annotated using GPL571. The average value of multiple probes corresponding to the same gene was taken as its expression value.

**Table 1:** Gene expression datasets used in this study.

GEO ID	Samples (PD: N)	Platform	Year	Author	Type
GSE6613	50:22	GPL96	2006	Scherzer CR	Whole blood
GSE72267	40:19	GPL571	2015	Roncaglia P	Whole blood

PD = Parkinson disease.

### 2.2. Immune-related gene extraction and differential expression analysis

From the immunology database, 1793 immune-related genes (IRGs) were retrieved and downloaded. Differential expression analysis between PD and control groups was performed with microarray expression data using the limma software package. The screening criteria for DEGs were set at *P* < .05, |log2 FC|>0.1, and were visualized using volcano plots. The IRGs with differences were filtered out and the results were displayed by a heat-map.

### 2.3. WGCNA

We used the WGCNA package to build networks and visualize them. When we constructed the network, we selected the soft-threshold power as the minimum power, and its scale-free topology fitting index reached 0.90. The minimum module size is set to 30. Then, the adjacency matrix was calculated according to the kernel value, and the adjacency matrix is converted into a topological overlap matrix (TOM) and the corresponding dissimilarity matrix (1-TOM). We clustered the genes by using 1-TOM and then built a dynamic pruning tree to identify the modules, by setting the merging threshold function at 0.25.

In order to identify the pivotal modules related to PD, we calculated the ME of each module using the “moduleEigegenes” function, and use the “Pearson” method to analyze the correlation with PD, we selected the module with the highest correlation with PD as the hub module. Genes within the hub modules are selected based on their connectivity and clinical trait relationships. Pearson correlations between genes and clinical traits were used to determine the clinical trait relationship (GS). Based on the criteria of |GS|>0.2 & |MM|>0.7, the genes in this module were screened, and candidate hub genes were obtained.

### 2.4. Construction and validation of diagnostic models

We obtained the hub gene by intersecting the differential gene and the candidate hub gene. In the experimental group GSE6613 data, to reduce the number of genes in the risk model, the Lasso (Least absolute shrinkage and selection operator, Tibshirani [1996]) method was used. Finally, the immune-related diagnostic model was constructed using a multivariate Cox regression model and feature genes were used to construct the diagnostic model. Receiver operating characteristic (ROC) analysis was performed using the R package “pROC (version 1.15.3, https://github.com/xrobin/pROC)” to calculate the area under the curve (AUC). AUC ranges from 0 to 1, where 0.7 is acceptable performance and 0.9 is excellent. The accuracy of this model and the expression consistency of the eigengenes were verified in the validation dataset GSE72267.

### 2.5. Functional enrichment analysis

By using the Gene Ontology database, an enrichment analysis of gene ontology terms was performed. Moreover, Fisher exact test was used to determine which items or pathways were significantly related to a set of genes in the Kyoto Encyclopedia of Genes and Genomes (KEGG) pathway enrichment analysis.

### 2.6. Protein-Protein Interaction (PPI) Network

PPI was performed via the STRING (https://stringdb.org/) database. The confidence of protein interaction was set as combined score > 0.4. Then, the Cytoscape software (https://cytoscape.org/) was utilized to visualize the PPI network.^[11]^ In the PPI network, hub genes were determined based on the degree of connectivity of the nodes.

### 2.7. Drug-hub gene interaction

We selected 10 genes with high degree in the constructed PPI network. The screened hub genes were also considered to be ideal targets for searching drug by the Drug-Gene Interaction database (DGIdb) (http://dgidb.genome.wustl.edu/). Drugs supported by no <2 databases or PubMed literature were validated as drug candidates. The final list includes only FDA-approved drugs. In addition, a network of identified target genes was constructed through the software STITCH database (http://stitch.embl.de/), which also contains drug-gene relationships.

## 3. Results

### 3.1. Identification of DEGs from the GEO database

**Figure 1.**
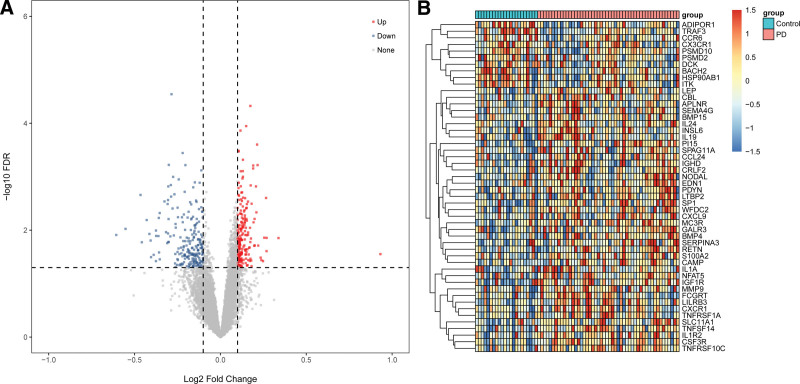
DEGs of GSE6613. (A) The volcanic map of DEGs. Red represented upregulated genes and blue represented downregulated genes. (B) The heat-map image of IRGs. Red represented up-regulation and blue represented down-regulation. DEGs = differentially expressed genes, IRGs = immune-related genes.

With the screening criteria of *P* < .05 and *|*log2 (FC)*|>*0.1, DEGs between PD and normal samples were identified. In the GSE6613 dataset, there were 220 upregulated and 216 downregulated genes between the whole blood of PD and normal samples. Among the differentially expressed genes, 50 DEGs were IRGs, of which 40 genes were upregulated and 10 genes were downregulated. The results were shown in Figure 1A and 1B.

### 3.2. WGCNA analysis identified immune-related Hub DEGs

**Figure 2.**
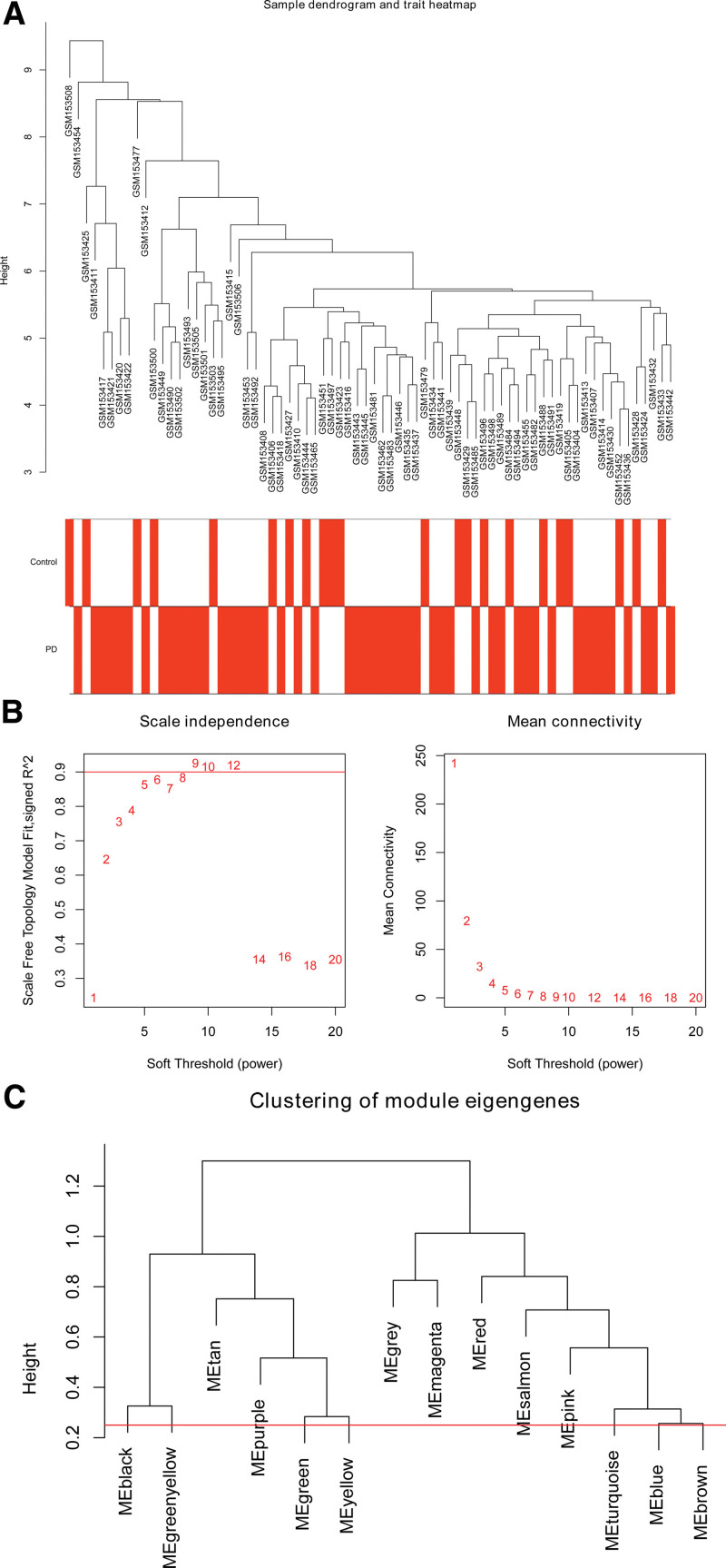
Construction of weighted gene correlation network. (A) Sample dendrogram and trait heat-map. (B–C) The relationship between scale-free topology model fit or mean connectivity and soft-threshold power.

**Figure 3.**
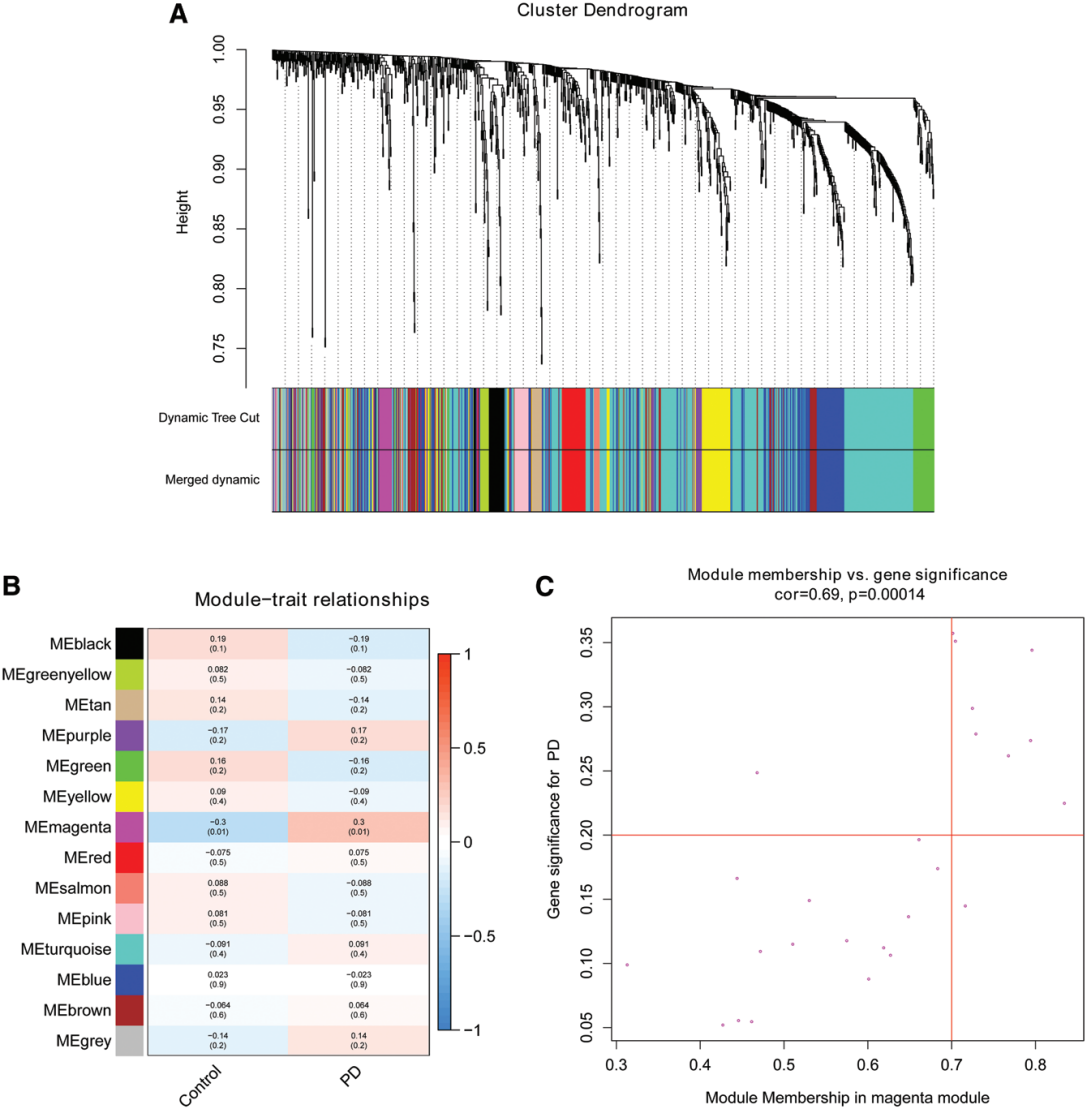
Weighted gene correlation network analysis. (A) Clustering dendrogram of genes based on topological overlapping. Different colors were assigned to corresponding modules. (B) Heat-map of associations between module eigengenes of normal and PD. (C) Candidate genes were screened in the magenta module. PD = Parkinson disease.

WGCNA analysis was performed on analyze the expression values of 1134 immune-related genes in 72 samples. The soft-threshold power for constructing gene regulatory networks is based on a scale-free R^2^ = 0.9, as shown in Figure 2. In Figure 3, based on average linkage hierarchical clustering and soft-threshold power, thirteen modules were identified. Among these 13 modules, the magenta module showed the highest correlation with PD (Pearson *R* = 0.3, *P* = .01), which considered as a hub module. The magenta module contained 25 IRGs and 8 genes remained after screening with the criteria of |GS|>0.2 & |MM|>0.7.

### 3.3. Construction and validation of Parkinson diagnostic models

**Figure 4.**
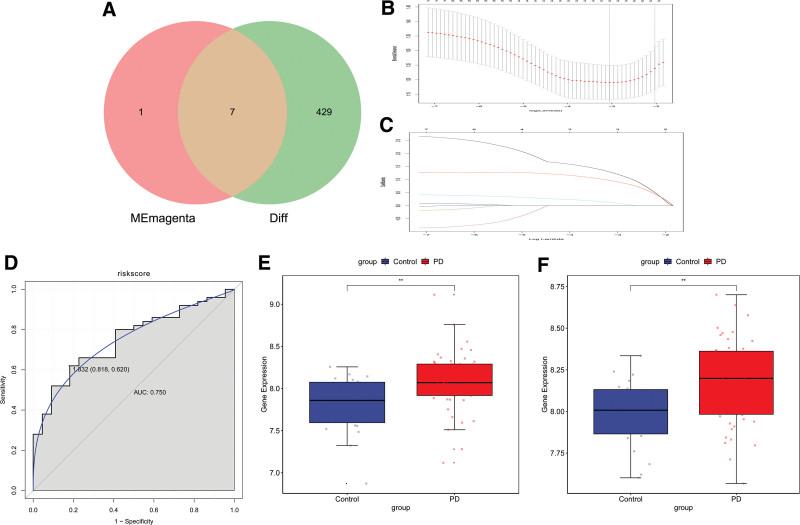
Construction of a diagnostic model for Parkinson disease. (A) Venn diagram of intersection of differential genes and candidate Hub genes. (B–C) LASSO Logistic regression based on GLMNET software package. (C) ROC of the diagnostic model in the training group. (E–F) expression of signature genes in the training group.

**Figure 5.**
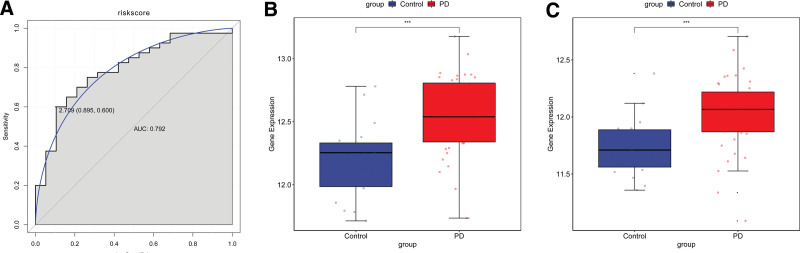
Validation of diagnostic models for Parkinson disease. (A) ROC of diagnostic model in validation group. (B–C) Expression of signature genes in the validation group.

We obtained 7 hub genes by intersecting the DEGs and candidate hub genes, as shown in Figure 4A. The GSE6613 dataset was screened using the Lasso method to reduce the number of genes in the risk model, as shown in Figure 4B and C. Among the 7 hub genes, there are 2 immune-related differential genes that can be used as diagnostic markers. We established the following formula using diagnostic features from the GSE6613 cohort: Risk Score = (0.07*LILRB3) + (0.15*CSF3R). We also used the ROC curve to evaluate the accuracy and specificity of the risk model, and it showed a diagnostic efficiency of 0.75, and the expression of both characteristic genes was upregulated, as shown in Figure 4D–F. And in the validation group GSE72267, the AUC area of this model reached 0.792, and the expression of 2 characteristic genes were also upregulated, as shown in Figure 5.

### 3.4. ceRNA network

**Figure 6.**
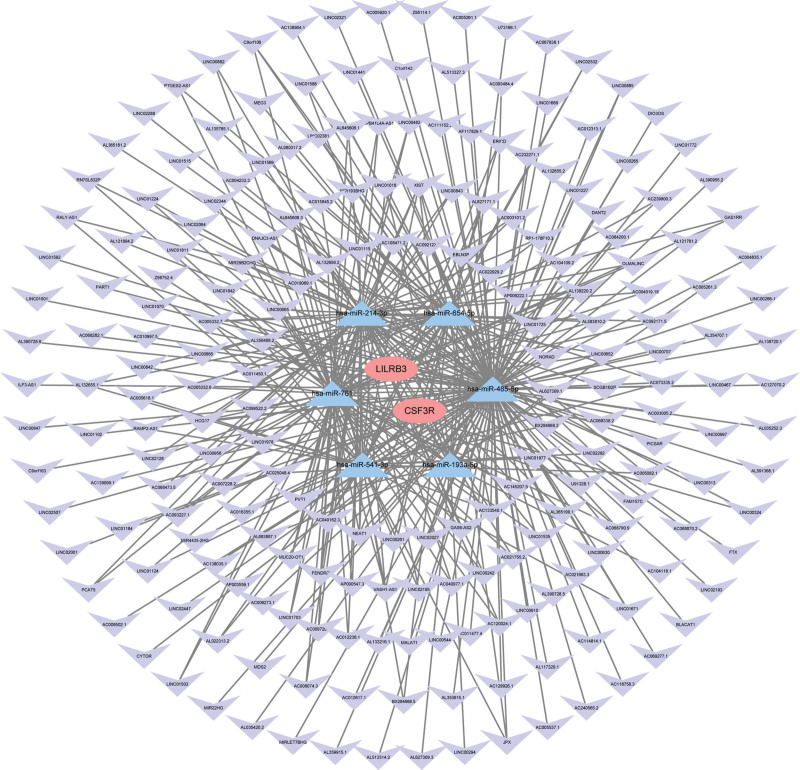
ceRNA network constructed by mRNA, miRNA and lncRNA.

In Figure 6, we built the ceRNA network to explore the regulatory relationship between hub genes (LILRB3 and CSF3R) and miRNA, lncRNA. In total, 6 miRNA nodes, 212 lncRNA nodes, and 152 edges were identified as differentially expressed profiles. These 2 signature genes represented in red, together with 6 miRNAs represented in blue and 212 lncRNAs represented in purple jointly constructed this multifactorial regulatory network.

### 3.5. Functional enrichment analysis of DEGs

**Figure 7.**
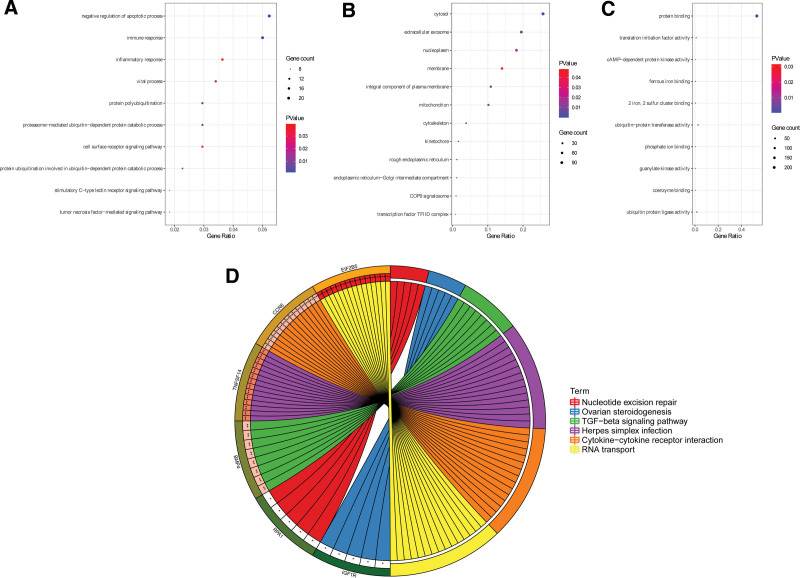
GO and KEGG enrichment analysis results of DEGs for PD. GO terms included biological process (BP), cellular component (CC), molecular function (MF) and KEGG. DEGs = differentially expressed genes, GO = gene ontology, PD = Parkinson disease.

David database was used to conduct functional enrichment analysis to investigate the biochemical processes and pathways involved in PD occurrence and development. The DEGs in PD were predominantly related to biological processes associated with PD such as negative regulation of apoptotic process, immune response, inflammatory response (Fig. 7A). Several key cellular components, including cytosol, exosomes, and nucleoplasm, were regulated by these DEGs (Fig. 7B). In addition, they had several key molecular functions, such as molecular functions, mainly protein binding, ubiquitin-protein transferase activity and ubiquitin-protein ligase activity and other protein-related functions (Fig. 7C). Based on results from the KEGG enrichment analysis, PD-related pathways are significantly enriched such as RNA transport, Herpes simplex infection, Cytokine-cytokine receptor interaction, TGF-beta signaling pathway, Nucleotide excision repair and Ovarian steroidogenesis (Fig. 7D).

### 3.6. PPI network analysis

**Figure 8.**
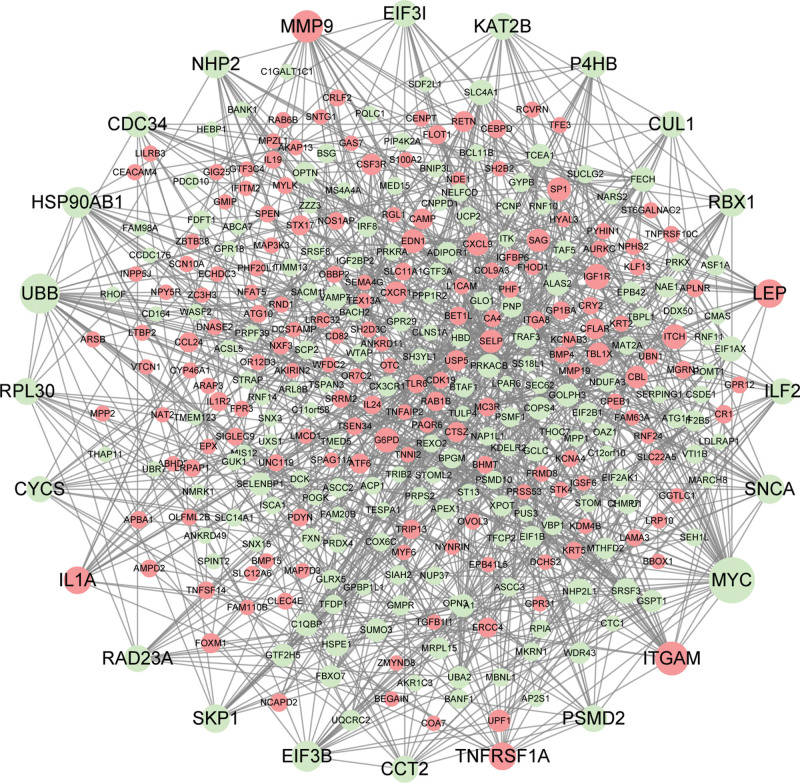
PPI networks for DEGs in PD. DEGs = differentially expressed genes, PD = Parkinson disease.

A PPI network was constructed using Cytoscape and the STRING database to investigate the relationships among DEGs. There were 300 nodes and 919 edges in the PPI network, we screened and obtained 24 genes with the criteria of degree ≥ 15, of which red represents upregulated genes and green represents downregulated genes, as shown in Figure 8. In addition, 4 of the top genes with relatively high connectivity degrees were: MYC (degree = 41), UBB (degree = 33), ITGAM (degree = 25) and MMP9 (degree = 23).

### 3.7. Drug-hub gene interaction

**Figure 9.**
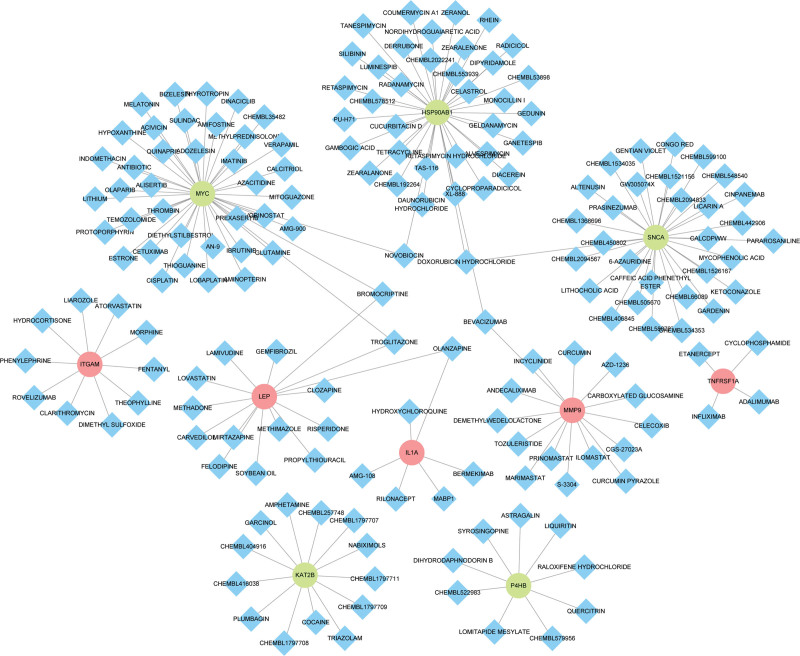
Constructed network of drug-gene interaction in Cytoscape. Red points represent upregulated genes, green points represent downregulated genes and blue points represent drugs.

Candidate genes were explored using DGIdb to examine potential molecular compounds or drugs that could alter their expression. Totally, we obtained 182 drug-gene interaction pairs in DGIdb, including genes (MMP9, IL1A, SNCA, LEP, TNFRSF1A, ITGAM, MYC, HSP90AB1, KAT2B, and P4HB) and 182 drugs. Figure 9 shows 41 drugs or molecular compounds that differentially regulate MYC expression. These include novobiocin, cetuximab, and verapamil. In addition, 38 drugs or molecular compounds, for instance, silibinin, diacerein, and zeranol, were found to interact with HSP90AB1. Furthermore, 31 drugs or molecular compounds regulated SNCA and 15 drugs or molecular compounds regulated MMP9.

## 4. Discussion

PD is a serious neurological disease. The gradual loss of substantia nigra dopaminergic neurons is one of the main causes of PD.^[5]^ Since PD has a long incubation period, patients are not aware of their early symptoms, resulting in patients being treated in the middle and late stages, not during the optimal time for treatment.^[12]^ Therefore, PD’s interconnected mechanisms need to be explored urgently. Herein, the WGCNA analysis provided a deeper understanding of PD-related molecular mechanisms by identifying critical genes and pathways. In this study, 7 hub genes (LILRB3, CSF3R, FCGRT, CXCR1, MMP9, TNFRSF1A, and IL1R2) were screened out by WGCNA. As potential diagnostic markers, LILRB3 and CSF3R were found to be immune-related differential genes. These key genes had potential regulatory effects on granulocyte proliferation and differentiation, immune response, pro-inflammatory activity and immune checkpoint factors.^[13,14]^ Previous studies have identified some biomarkers of PD, but have no immune-related biomarkers. Songyun Zhao et al^[15]^ identified 3 cuprotosis-related genes ATP7A, SLC31A1, and DBT associated with immune cells or immune function in PD and more accurate for the diagnosis of Parkinson disease course. Na Xing et al^[16]^ explored the combination model composed of LPIN1 and TNFAIP3, and each biomarker may serve as an efficient tool for distinguishing PD from healthy control. Then, the authors found that PPBP, PROS1, and LCN2 were identified and validated to be related to PD and PPBP, LCN2 may potentially be biomarkers or therapeutic targets for PD in peripheral blood mononuclear cell.

Results showed that these 7 key genes were mainly enriched in immune response, inflammatory response, and Cytokine-cytokine receptor interaction. Researches on the role of immune system in PD has advanced over the past decade. A number of evidence support the development of chronic inflammatory events in the brain and biological fluids (CSF and serum) of Parkinson disease.^[17]^ According to the researchers, PD is characterized by early immune activation that changes dynamically with the progression of the disease, leading to the degeneration of neurons during the course of the disease.^[18]^ Additionally, the innate and adaptive immune systems are activated during this inflammation, both in the brain and peripherally.^[17]^ Several neurodegenerative diseases, including Parkinson, are associated with gene dysfunction. This immune disorder results in a subsequent inflammatory response that stimulates the secretion of cytokines and chemokines by microglia or other immune cells, disturbs the proportion of lymphocyte subsets in peripheral blood, and leads to apoptosis of dopaminergic neurons.^[19]^ Genetic variants also cause abnormal immune-related signaling pathways, which lead to chronic inflammation and disruption of the blood-brain barrier, the nerve cells in the CNS are exposed to various molecules and blood cells, causing the neurons in the DA to be toxic.^[20]^ Therefore, while Parkinson disease progresses, inflammatory responses in the central nervous system contribute to accumulation and diffusion of synuclein in DA neurons. Therefore, in disease assessment, abnormal immune responses mediated by PD genes may be evaluated based on multiple immune molecules and inflammatory factors, which may be as potential biomarkers for PD patients.

MMP9, a member of the protein endopeptidase family, is an enzyme that helps remodel tissues by degrading extracellular matrix components.^[21]^ In the brain, MMP9 is expressed in multiple cell types, and pre-MMP9 precursors are released from cells that are activated extracellularly.^[22]^ In cerebrovascular diseases such as Alzheimer disease, elevated levels of MMP9 have been shown to be associated with various neurological and inflammatory disease states,^[23–25]^ cerebral amyloid angiopathy,^[26]^ ischemia,^[27]^ and cerebral hemorrhage.^[28]^ L1R2 expresses the receptor for IL1. The relationship between IL1 and dopaminergic dysfunction has been demonstrated. The level of IL1-b in serum of PD patients was higher than that of controls.^[29]^ The IL1B gene is associated with restless legs syndrome.^[30]^ Elevated levels of IL1 have also been observed in depressed patients.^[31]^ It suggests that immune factors may be involved in the pathogenesis of PD.

Several diseases are associated with dysregulation of the human leukocyte immunoglobulin-like receptor family, which was discovered more than 20 years ago.^[32]^ The LILRB molecule is considered as an immune checkpoint that controls and limits overt immune responses.^[33,34]^ Among inhibitory LILRB molecules, A relatively restricted and highly expressed expression of LILRB3 in myeloid cells makes it an attractive immunomodulatory target due to its 4 extracellular Ig-like domains and 4 cytoplasmic ITIMs.^[33,34]^ However, its exact functional and immunomodulatory potential has not been fully exploited due to the lack of specific reagents and model systems. The CSF3R receptor binds to granulocyte colony-stimulating factor, a crucial cytokine for granulocyte proliferation and differentiation.^[35]^ New biomarkers associated with diagnosis were identified in this study: LILRB3 and CSF3R, 2 immune-related genes. The diagnosis model established in this study showed high diagnostic value and accuracy through various analyses.

In this study, hub genes (ITGAM, MMP9, LEP, IL1A, TNFRSF1A, MYC, HSP90AB1, SNCA, KAT2B and P4HB) in the PPI networks were identified in PD. Based on these hub genes, The DGIdb database revealed 182 drug-gene interactions, including 41 drugs and molecular compounds that differentially regulated the expression of MYC. HSP90AB1 has also been found to interact with 38 drugs or molecular compounds, such as silibinin, diacerein, and zeranol. Furthermore, 31 drugs or molecular compounds regulated SNCA and 15 drugs or molecular compounds regulated MMP9. NCI, drug banks, and other drug-related databases provide all data resources. Inhibitors and agonists are the primary relationship between these drugs and genes.

Cinpanemab is a humanized monoclonal antibody. It is generated from memory B cells from individuals without any neurological disease using Biogen clonal selection technology. In addition to targeting SNCA, it exhibits antibody-like properties, making it a promising treatment for Parkinson disease. SNCA is a major risk gene for PD, and gene polymorphisms of SNCA are associated with the common sporadic form of PD.^[36]^ Mouse model was inoculated with α-synuclein preformed fibrils to observe the effect of Cinpanemab on PD.^[37,38]^ Weekly intravenous injection of Cinpanemab resulted in a marked delay in the onset of first paralysis for 7 days and weight loss for 9 days. This information demonstrated that Cinpanemab could delay paralysis and motor symptoms associated with alpha-synuclein proliferation. The next experiment was to test the effect of Cinpanemab on motor function. After 60 days of Cinpanemab treatment, the motor function was observed by wire suspension test and found that the treated mice performed significantly better and took longer to fall off the wire than the drug-treated mice.^[39]^ In conclusion, Cinpanemab treatment resulted in reduced truncated α-synuclein 6 kDa variants and enhanced motor function.

Prisnezumab targets a carboxyl terminus on human alpha-synuclein and is a monoclonal antibody. Transgenic mice were used to evaluate the efficacy and potency of prasinezumab in preclinical studies.^[39,40]^ Prasinezumab or other alpha-synuclein antibodies were administered weekly intraperitoneally to the mice. A reduction in neuronal and synaptic loss was observed, as well as a reduction in gliosis and an improvement in cognitive and motor functions.^[40]^ These preclinical studies suggest that prasinezumab may slow the course of Parkinson disease. Although the analysis method in this article can identify immune-related genes in the characterization of Parkinson progression, there are some limitations in this study. Firstly, these results were obtained in a small group of participants. Due to the inconvenience and difficulty of conducting long follow-up studies, more detailed clinical information and scale assessment data cannot be obtained. Comprehensive clinical information is conducive to the objective evaluation of research results, and we will strengthen the acquisition of clinical data of patients to enable subgroup analysis in the future.

## 5. Conclusion

In conclusion, we obtained a novel diagnosis model for PD, which included 2 immune-related key genes LILRB3 and CSF3R. These crucial genes may influence the immune function of tumor-infiltrating immune cells. An augmented immune response and appropriate immune regulation may contribute to PD prognosis. The gene ontology and KEGG analysis of the selected genes provided a deeper understanding of PD development. Furthermore, this study provides substantial additional information for future research on PD pathogenesis and targeted therapies.

## Author contributions

**Conceptualization:** Xiaoxia Yang.

**Data curation:** Zhiyun Wang.

**Formal analysis:** Zhiyun Wang.

**Funding acquisition:** Xiaoxia Yang.

**Writing – original draft:** Zhiyun Wang.

**Writing – review & editing:** Xiaoxia Yang.
